# Relationship between single nucleotide polymorphism of NOS2 gene and inheritance of allergic rhinitis in children

**DOI:** 10.3389/fgene.2023.1126212

**Published:** 2023-02-08

**Authors:** Xionghui Wu, Sijun Zhao, Weiqing Huang, Min Huang, Jiang Xie, Guangliang Liu, Shuting Chang

**Affiliations:** ^1^ Department of Otorhinolaryngology Head and Neck Surgery, Hunan Children’s Hospital, Changsha, Hunan, China; ^2^ Department of Neonatology, Hunan Children’s Hospital, Changsha, Hunan, China

**Keywords:** allergic rhinitis, gene single nucleotide polymorphism, genetic analysis, intelligent medical treatment, gene and inheritance of allergic rhinitis

## Abstract

Allergic rhinitis is a common chronic disease, and its high incidence has a great negative impact on the quality of life of many people, especially children. In this paper, through in-depth analysis of NOS2 gene polymorphism, the protective mechanism of NOS2 gene against AR was studied to provide theoretical and scientific basis for the diagnosis of children with AR. It was concluded that the concentration of Immunoglobulin E (IgE) in rs2297516 was 0.24 IU/mL compared with that in normal children. rs3794766 specific IgE concentration in the children group was increased by 0.36 IU/mL, which was higher than that in the healthy children group; the difference of rs7406657 specific IgE concentration between the children group and the healthy group was 0.03 IU/mL. The total serum IgE concentration in the healthy children group was lower than that in the infant group, and the change of Rs3794766 was the least, followed by rs2297516 and rs7406657. This means that rs7406657 is the highest, rs2297516 had general genetic correlation with AR patients, and rs3794766 had the least genetic correlation with AR patients. Among the three groups of SNP loci, the healthy children group was higher than the patient children group, indicating that AR reduces the gene frequency of the three loci, and the reduction of gene frequency will also increase the susceptibility of children to AR, because the frequency of gene occurrence will affect the gene sequence. In conclusion, smart medicine and gene SNPS can promote the detection and treatment of AR.

## 1 Introduction

Rhinitis is widely defined as inflammation of nasal mucosa, which is a common disease affecting about 40% of the population. AR is a common chronic rhinitis, accounting for 10%–20% of the world population. Severe AR may have more adverse effects on quality of life, sleep and work, because severe AR leads to respiratory obstruction in children. Intelligent medical treatment can improve the detection of influencing factors of AR, and detect AR related genes through intelligent devices, thus improving the detection accuracy. AR not only makes life difficult for adults, but also affects the quality of life of many children. Therefore, the study of AR has important clinical and social significance.

AR can affect respiratory tract infection in children. Bousquet Jean put forward the first guide based on recommendation grading evaluation and development evaluation, integrating the application of supporting research in AR management. The purpose was to improve the accuracy of the diagnosis strategy and treatment plan in allergic rhinitis (Bousquet et al., 2020a). Small Peter believed that allergen immunotherapy was an effective immunomodulation therapy. If the drug treatment of allergic rhinitis was ineffective or intolerable, or the patient chooses, it should be recommended (Small et al., 2018). Schuler IV Charles Frank studied the influencing factors and causes of children’s AR, and proposed some coping strategies (Schuler et al., 2021). Okubo Kimihiro believed that it was necessary to understand AR through a guide and use this knowledge to develop treatment plans. He also discussed the concept of AR and cost-benefit analysis (Okubo et al., 2020). Scadding Glenis K believed that the combined treatment of intranasal corticosteroids and intranasal antihistamines was more effective than any one alone, which could provide second-line treatment for rhinitis patients with poor control after single drug treatment (Scadding et al., 2017). Meng Yifan covered recent studies on SNP, DNA methylation, regulatory B cells, immunotherapy and the role of biological agents in AR (Meng et al., 2019). Zhang Yuan needed to better understand the prevalence and characteristics of AR, sensitization mode and related risk factors, so as to improve treatment and develop effective allergic rhinitis prevention strategies (Zhang and Zhang, 2019). The above studies all described the hazards and influencing factors of AR, but did not combine with the SNP of the gene.

SNP is closely related to AR. Chen Min-Li studied the relationship between SNP and AR through meta-analysis of SNP and AR risk in interleukin-13 and differentiation 14 gene clusters (Chen et al., 2018). Falahi Sara used polymerase chain reaction restriction fragment length polymorphism to determine the relationship between SNP of interleukin-33 gene and AR (Falahi et al., 2022). Amarin Justin Z used binomial logistic regression to study the association based on genotype under the general, recessive and dominant models of disease penetrance, and believed that the marker selection in the future genetic association study of asthma and allergic rhinitis should include functional polymorphism (Amarin et al., 2017). Ke Xia aimed to evaluate the potential association between gene SNP and AR in Han population. His results showed that it was significantly related to AR risk in Han population (Ke et al., 2017). The above studies all described the relationship between SNP and AR, but there are still some deficiencies in genetic research.

The treatment plan for AR can effectively relieve the symptoms of patients, which is usually safe and positive. However, it still cannot meet people’s expectations for the treatment of diseases, because the current treatment plan still cannot completely cure allergic rhinitis. Even after treatment, the quality of life of patients still suffers from some adverse effects. Intelligent medical treatment can improve the detection of influencing factors of AR, and detect AR related genes through intelligent devices, thus improving the detection accuracy. Therefore, in the aspect of AR treatment, it is necessary to discuss the treatment mechanism and pathogenesis in detail. By measuring the concentration of total IgE in patients’ serum, the degree of association between allergic factors and NOS2 gene polymorphism is analyzed.

## 2 Factors of AR genetics

### 2.1 Etiology and genetic factors of AR

**FIGURE 1:**
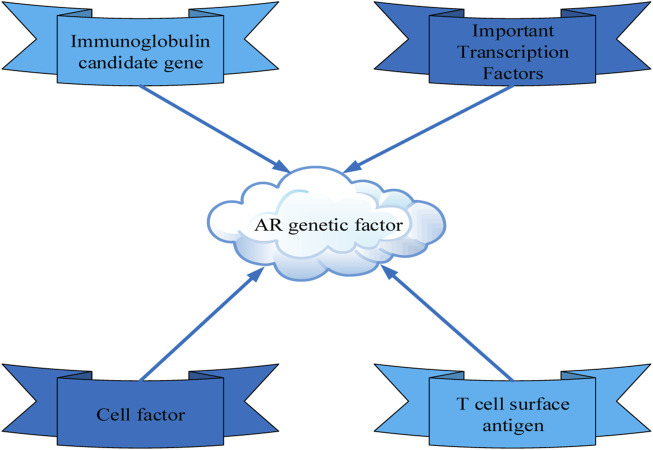
Genetic factors of AR.

AR is a multifactorial disease caused by the interaction between gene and environment. Genetic research shows that AR is a complex genetic disease, in which environmental factors are mainly related to various allergens in the human environment (Numminen, 2017). Many symptoms of AR are highly genetically controlled and are polygenic. Allergic rhinitis is affected by a variety of allergens. AR genetic factors are a variety of disease related genes and their related transcription factors, involving the selection of immunoglobulin candidate genes, important transcription factors, cytokines, T cell surface antigens and other pathogenic genes, as shown in Figure 1. From the genetic point of view, the impact of environmental factors on allergic rhinitis can be determined. In addition, environmental changes allow the innate and acquired immunity of the respiratory mucosal immune system to be regulated through various genetic mechanisms, thereby increasing the vulnerability of patients to allergic genes.

### 2.2 Inducers of AR

**FIGURE 2:**
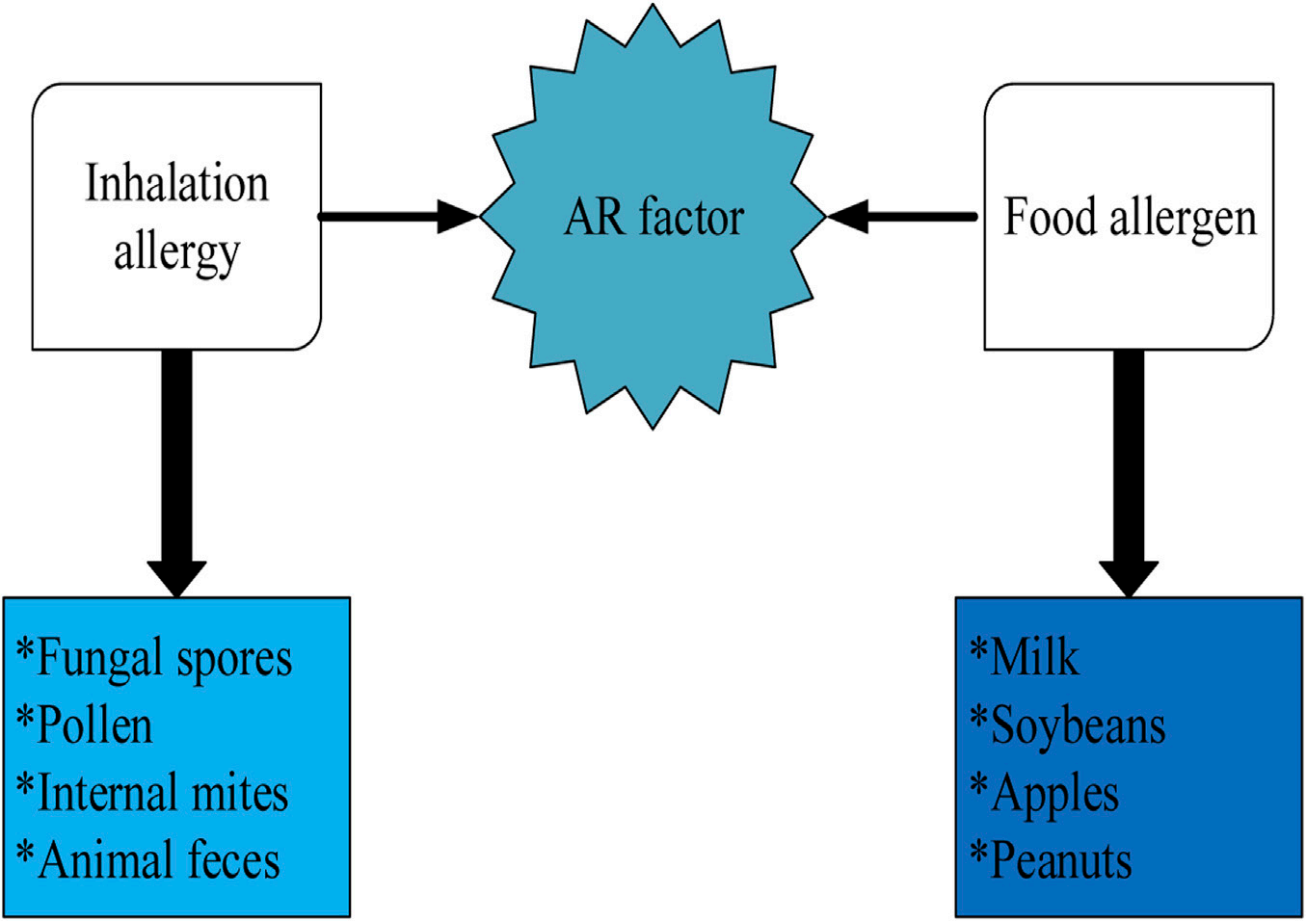
Inducement factors of AR.

Figure 2 shows the specific IgE antibody reaction caused by environmental allergens (mainly inhalation allergens and food allergens), among which inhalation allergens are the main cause of AR (Blaiss et al., 2018). Inhalation allergens include fungal spores, pollen, internal mites, animal feces, etc. Their concentrations have a great relationship with the severity of allergic respiratory symptoms. The higher the concentration of allergen, the greater the probability of infection. Allergens cause skin allergy, gastrointestinal and nasal symptoms, but rarely AR. For infants, food allergy is mainly related to milk and soybeans. Common food allergies in adults include peanuts, nuts, fish, eggs, milk, soybeans, apples, pears, etc. In addition, it should be noted that allergies to certain vegetables and fruits may cause cross reactions with plant pollen.

### 2.3 Treatment of AR

**FIGURE 3:**
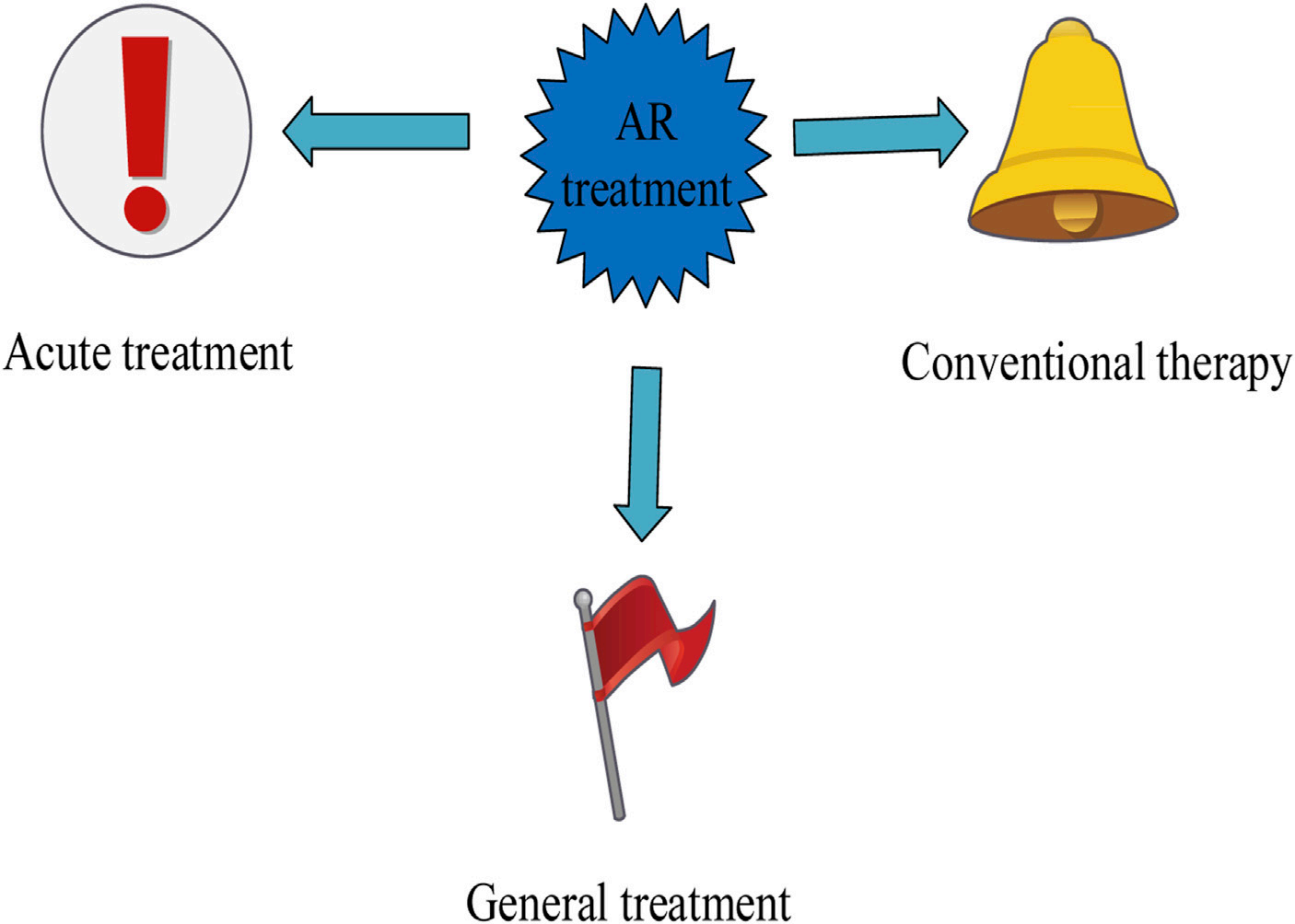
Treatment of AR.

The treatment principle is to avoid contact with allergens, reasonably use antihistamines and glucocorticoids, and give special immunization to patients. In addition, IgE monoclonal antibody is very effective for severe AR, but it is expensive, because the preparation process of monoclonal antibody is complex. Active and effective AR treatment can prevent and reduce asthma attack. Although AR has not been completely cured, the standardization of combined treatment is helpful to optimize symptom control and significantly improve the quality of life of patients. The treatment of AR includes acute treatment, routine treatment and drug treatment, as shown in Figure 3. When the patient’s rhinitis obstructs nostril breathing or shock occurs, a doctor should be immediately arranged for emergency treatment. General treatment includes avoiding contact with allergens, especially identified allergens, and avoiding contact as much as possible. People with pollination allergy cannot walk during pollination. In addition, tools can be used to fight allergies, reduce nasal inhalation or allergic contact with the shell, and reduce nasal and eye symptoms. Allergies to indoor microorganisms and mites should be ventilated regularly to keep the room clean and dry. People who are allergic to dandruff, feathers and feces should avoid contact with animals as much as possible. The characteristics of drug therapy are very different, lacking the best, fast and effective drug use methods. In addition to ordinary generic drugs, under the guidance of doctors, full consideration must be given to personal conditions to select the most appropriate drugs, mainly through the appropriate dosage for targeted treatment. In addition, drug therapy can greatly shorten the treatment time and achieve rapid results (Bousquet et al., 2020b).

### 2.4 Application of intelligent medical treatment in AR

The current intelligent medical treatment can use ultrasound and radio frequency for AR treatment. In particular, ultrasound therapy is mainly used for focused ultrasound technology, rather than invasive technology. It allows the ultrasound generated outside the body to focus on specific human targets, and the focus area has high energy. The biological effect of ultrasound on nasal submucous tissue can reduce the sensitivity of nerves, blood vessels and clay glands. It can immediately change the structure and function of target tissues to reduce the hypersensitivity reaction of nasal mucosa, thus increasing the effective nasal respiratory area. By selectively destroying parasympathetic nerve fibers, AR can significantly improve nasal itching, sneezing, nasal mucus and other diseases, and improve the symptoms of patients (Tartarisco et al., 2017). Moreover, the idea of non-invasive treatment has become that there is no damage to the invasive tissues. The ultrasonic rhinitis treatment instrument uses non-invasive ultrasonic therapy to treat allergic rhinitis, and provides doctors with new options for effective treatment of complications because non-invasive ultrasound therapy is more convenient. High frequency therapy is a kind of high-frequency electromagnetic band. Among them, the magnetic field can spread directly in the atmosphere or vacuum without being affected by the conductor. Low temperature plasma is the most widely used RF device in clinical application. The conductive medium forms a high concentration plasma area around the electrode, which is composed of highly ionized particles. These ionized particles have enough energy to tear the organic molecular chains in the tissue, separate molecules and reduce the tissue volume. The advantages of high-frequency treatment are relatively small damage to surrounding tissues, low treatment temperature, ablation of tissue targets, low thermal conductivity, less free radical release, less inflammatory reaction, and no carbon and environmental pollution.

## 3 Experimental data and methods of genetic association between NOS2 gene SNP and children AR

### 3.1 Clinical data

According to AR diagnosis and treatment methods, 285 children with AR are selected from the otorhinolaryngology department of Hunan Children’s Hospital, P.R. China, from December 2020 to December 2021, including 167 boys and 118 girls, aged from 4 to 14 years. Then, 352 healthy children are searched again, including 157 boys and 195 girls, who are divided into two groups: the patient children group and the healthy children group. All children in the patient’s children group have many symptoms, such as nasal congestion, poor breathing, sneezing and nose itching. Allergen skin prick test (ASPT) is AR positive, and each patient has a history of allergy inheritance. All children in the healthy children group have no genetic history of allergy, and ASPT is AR negative. In addition, all children in this experiment have no other allergic diseases and allergic genetic history, and the accuracy of the experiment is improved by excluding other criteria.

### 3.2 Experimental methods

1) The ASPT experiment and grading are carried out according to the normal classification standard, and the ASPT grading of the patient group is ≥1.
2) The patient’s serum IgE is detected using intelligent detection equipment system.
3) All patients’ genomic DNA is extracted by elbow vein blood test and refrigerated with a unified anti coagulation test tube.
4) There are three kinds of SNP site selection: rs2297516, rs3794766, rs7406657.
5) The SNP classification uses an intelligent experimental platform for classification and detection. The chip with the sample is tested internally in the mass spectrometer, and the locus genotype of the gene sequence is detected with the support of intelligent equipment.

### 3.3 Statistical methods

Statistical analysis is carried out with data analysis software. The measurement data are expressed in the way of mean value. The gender difference between the two groups is compared with x^2^ Inspection. The degree of fit is used to test whether the gene distribution conforms to Hardy Weinberg equilibrium. x^2^ is used to test the correlation between allele frequency, haplotype, multilinear regression analysis SNP and total serum IgE, and modify according to age and sex to determine the main genetic model of A<0.05.

## 4 Experimental results of genetic association between NOS2 gene SNP and children’s AR

First, the gender difference between the two groups of children in the genetic association analysis of NOS2 gene SNP and children’s AR is analyzed to determine whether the two groups of children met the test criteria. The specific differences are shown in Table 1.

**TABLE 1:** Analysis of gender differences between the two groups of children.

	Patient children group	Healthy children group
Boy	167	157
Girl	118	195
x^2^	0.251	0.025
A	0.458	0.001

**FIGURE 4:**
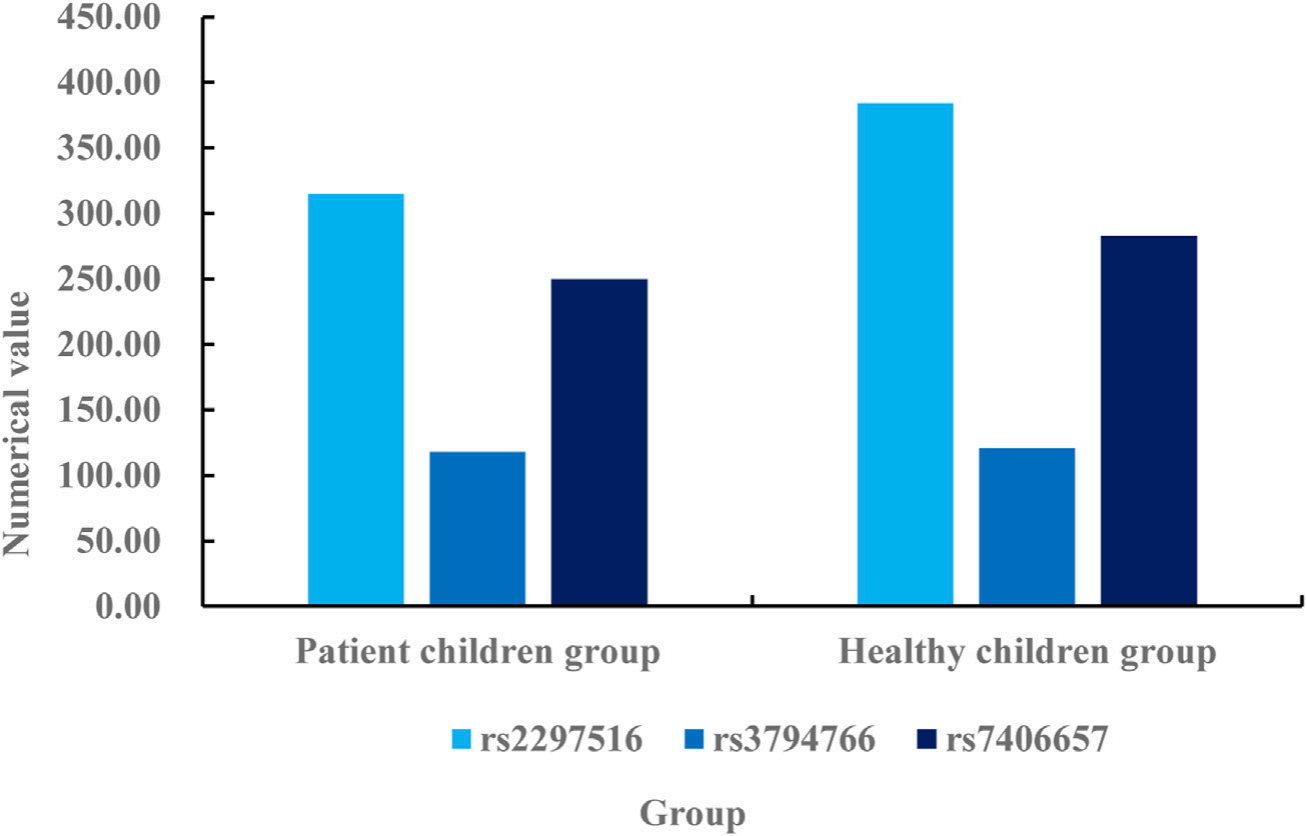
Comparison of allele frequency difference of NOS2 between two groups of children.

According to the data described in Table 1, there are 167 boys and 118 girls in the patient children group, with a difference of 49 men and women. There are 157 boys and 195 girls in the healthy children group, with a difference of 38 men and women. There are 10 differences between boys in the patient children group and boys in the healthy children group, and 77 differences between girls and girls in the healthy children group. The x^2^of the patient children group is 0.251, and the x^2^of the healthy children group is 0.025. The difference between the two groups is 0.226. The A of the patient children group is 0.458, and the A of the healthy children group is 0.001. The difference between the two groups is 0.457. Through the analysis and comparison of x^2^and A, it can be known that the gender differences between the two groups meet the statistical standards. Then, the allele frequency difference of NOS2 between the two groups of children is analyzed, mainly using single locus analysis. The specific analysis is shown in Figure 4.

According to the allele frequency difference of NOS2 between the two groups of children described in Figure 4, the allele frequency of NOS2 in the healthy children group is higher than that in the patient children group. It can be seen from the figure that the data of rs2297516 in the patient children group under single point detection is 315, while the data of rs2297516 in the healthy children group under single point detection is 384. The data of healthy children group is 69% higher than that of patient children group. The data of rs3794766 in the patient children group under single point detection is 118, while the data of rs3794766 in the healthy children group under single point detection is 121. The data of the healthy children group is 3 times higher than that of the patient children group. The data of rs7406657 in the patient children group under single point detection is 250, while the data of rs7406657 in the healthy children group under single point detection is 283. The data of the healthy children group is 33% higher than that of the patient children group. The SNP loci of the three groups are higher in the healthy children group than in the patient children group, which indicates that AR reduces the gene frequency of the three loci, and the decrease in gene frequency also leads to an increase in the susceptibility of children to AR. Then, the difference of the distribution of NOS2 gene SNP genotype between the patient children group and the healthy children group is analyzed. The specific comparison is shown in Table 2.

**TABLE 2:** Difference of distribution of NOS2 gene SNP genotype between patient children group and healthy children group.

SNP	Genotype	Patient children group	Healthy children group	A
rs2297516	AA	78	83	0.551
	CC	110	125	
	AC	175	214	
rs3794766	TT	5	9	0.974
	CC	269	308	
	CT	108	101	
rs7406657	CG	135	179	0.084
	CC	48	44	
	GG	192	204	

**FIGURE 5:**
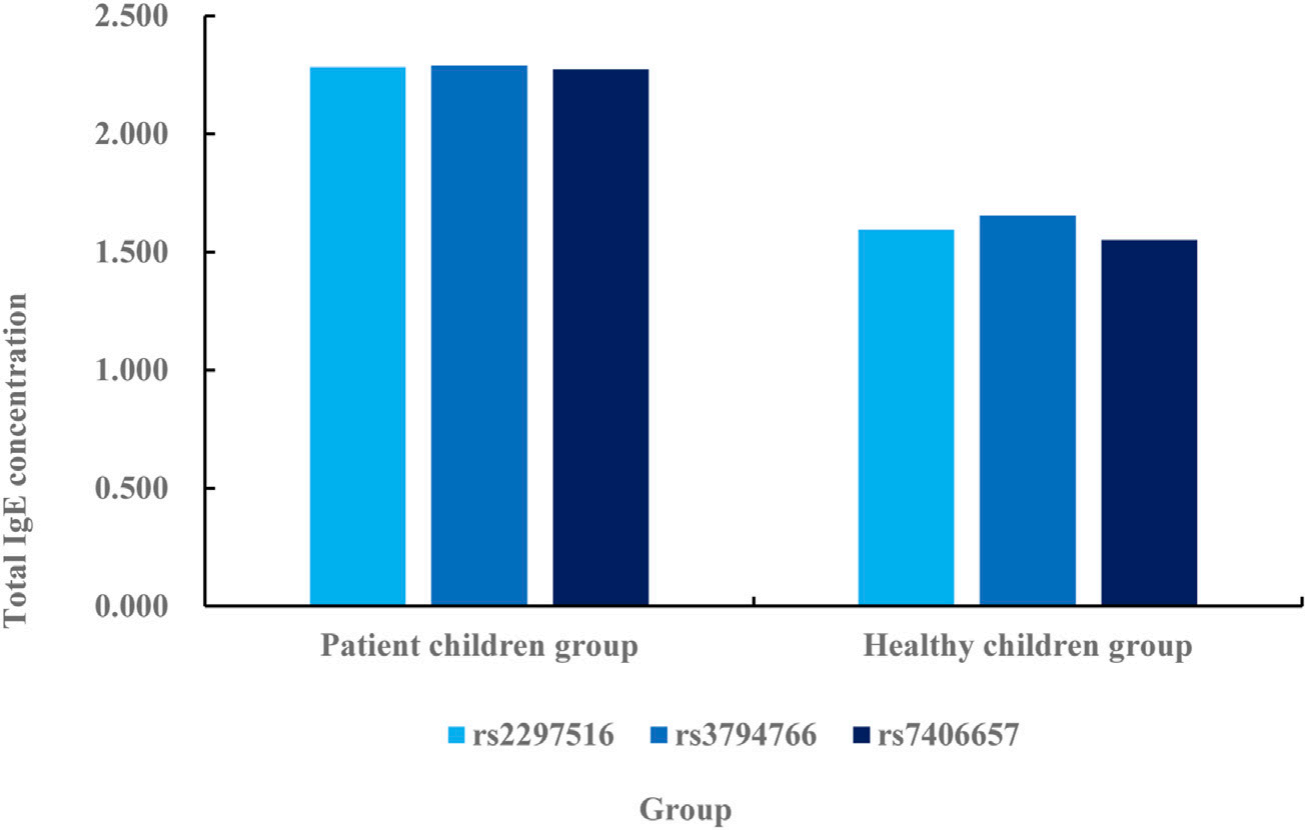
Correlation analysis between NOS2 gene SNP and total serum IgE concentration in patients and healthy children.

According to the data described in Table 2, rs2,297,516 genotypes are AA, CC and AC respectively. The AA, CC, and AC of the patients’ children group are 78, 110 and 175 respectively, while the AA, CC, and AC of the healthy children group are 83, 125 and 214 respectively. Compared with the two groups, the AA of the healthy children group is 5. CC of healthy children is 15% higher than that of patients. The AC of healthy children is 39. The distribution of these three genes is higher in the healthy children group than in the patient children group, and A is 0.551, indicating that the differences between the two groups meet the statistical standard. The distribution of rs3794766 genotype is TT, CC and CT respectively. TT is 5, CC is 269, and CT is 108 in the patient children group, while TT is 9, CC is 308, and CT is 101 in the healthy children group. Compared with the two groups, TT in the healthy children group is 4. The CC of healthy children is 39. The CT of the healthy children group is 7. The distribution of TT and CC genes in healthy children is higher than that in patients. However, CT shows that the healthy children group is lower than the patient children group, and A is 0.974, indicating that the difference between the two groups conforms to the statistical standard. The distribution of rs3794766 genotype is CG, CC, and GG. The CG, CC, and GG of the patient children group are 135, 48, and 192 respectively, while the CG, CC, and GG of the healthy children group are 179, 44, and 204 respectively. Compared with the two groups, CG of the healthy children group is 44. The CC of healthy children is 4. The GG of healthy children group is 12. The distribution of CG and GG genes in healthy children is higher than that in patients. CC is lower in the healthy children group than in the patient children group, and A is 0.084, indicating that the difference between the two groups meets the statistical standard. Then, the correlation analysis between NOS2 gene SNP and serum total IgE concentration in patients’ children group and healthy children group is analyzed. The specific changes are shown in Figure 5.

**FIGURE 6:**
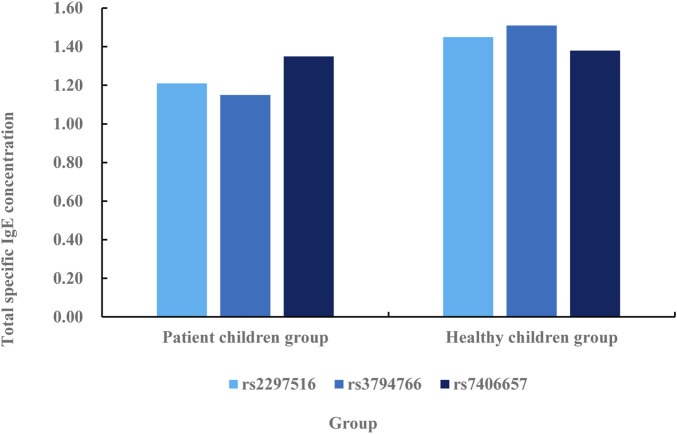
Association between NOS2 gene SNP and specific IgE.

According to the comparative data in Figure 5, the total serum IgE concentration in rs2297516 of the patient’s children group is 2.284 IU/mL, while that in rs2297516 of the healthy children group is 1.595 IU/mL, with a difference of 0.689 IU/mL. The total serum IgE concentration in rs3794766 is 2.291 IU/mL in children group, while that in rs3794766 in healthy children group is 1.654 IU/mL, with a difference of 0.637 IU/mL. The total serum IgE concentration in rs7406657 of the children group is 2.274 IU/mL, while the total serum IgE concentration in rs7406657 of the healthy children group is 1.551 IU/mL, with a difference of 0.723 IU/mL. The total serum IgE concentration of these three gene sequences is lower in the healthy children group than in the patient children group, and the change of rs3794766 is the smallest, followed by rs2297516, and finally rs7406657. This shows that rs7406657 has the highest genetic correlation with AR patients, while rs2297516 has a general genetic correlation with AR patients. The genetic correlation between rs3794766 and AR patients is the smallest. Finally, the association between NOS2 gene SNP and specific IgE is analyzed. The specific association analysis is shown in Figure 6.

According to the comparative data in Figure 6, the specific IgE concentration in rs2297516 of the patient’s children group is 1.21 IU/mL, while the specific IgE concentration in rs2297516 of the healthy children group is 1.45 IU/mL, with a difference of 0.24 IU/mL. The specific IgE concentration in rs3794766 is 1.15 IU/mL in the children group, while that in the healthy children group is 1.51 IU/mL, with a difference of 0.36 IU/mL. The specific IgE concentration in rs7406657 of the children group is 1.35 IU/mL, while the specific IgE concentration in rs7406657 of the healthy children group is 1.38 IU/mL, with a difference of 0.03 IU/mL. On the whole, the specific IgE concentration in the healthy children group is higher than that in the patient children group. This shows that healthy children are more specific to allergens and can be protected from the infection of these allergens. The children of the patients have low resistance to these allergens and are vulnerable to the infection of these substances. The total IgE concentration in the serum of the patient’s children is relatively low, which is unable to effectively respond to the invasion of allergens.

## 5 Discussion

AR is a disease determined by genetic and environmental factors, which plays an important role in the occurrence of AR. Special immunotherapy is a method to treat allergic diseases and the only way to change the natural process of AR, which is to improve patients’ allergen resistance by changing the role of target cells. At present, AR is widely considered as a genetic and environmental disease caused by genetic and environmental factors. SNP is a common genetic factor that affects people’s susceptibility to disease. Studies have shown that the sensitivity of antiretroviral drugs and the effectiveness of drug therapy are related to multiple gene SNPs. The DNA differences of different races, groups and individuals found in the research of human genome observation system and their importance fundamentally change the diagnosis, treatment and prevention of diseases. NOS2 gene is located on chromosome 17, 37 KB long, with 26 exons. The second external element has the starting point of the converter, and the 26th external element has the final code. It runs a subspace area with many built-in factors required to activate the initializer. NOS2 genotype has multiple overlapping open reading frames, and the activation of subspace may affect the expression of INOS. The study found that the rs2297516 data of the healthy children group is 69% higher than that of the patient children group under the single point detection. The rs3794766 data of the healthy children group is 3% higher than that of the patient children group. The rs7406657 data of the healthy children group is 33% higher than that of the patient children group. The total serum IgE concentration of these three gene sequences is lower in the healthy children group than in the patient children group, and the change of rs3794766 is the smallest, followed by rs2297516, and finally rs7406657. This shows that rs7,406,657 has the highest genetic correlation with AR patients. The rs2,297,516 has the general genetic correlation with AR patients, and rs3,794,766 has the lowest genetic correlation with AR patients.

## 6 Conclusion

In the research of human genome SNP observation system, the DNA differences have found in different races, groups and individuals and their important properties fundamentally have changed the diagnosis, treatment and prevention of AR diseases. The SNP of NOS2 gene may be related to children’s AR. The SNP genotype distribution of NOS2 gene analyzed in the experiment is very similar to the genetic sequence of allergic rhinitis. Intelligent medical treatment has improved the detection of influencing factors of AR, and AR related genes was detected through intelligent devices, thus improving the detection accuracy. In addition, NOS2 gene SNP has a certain correlation with the total serum IgE concentration. The potential role of NOS2 gene SNP in the formation and development of AR has required a large number of samples, multi regions and multi countries to conduct in-depth research, which has laid a theoretical foundation for developing new AR control direction. However, given the limited number of samples collected in this study, it is necessary to increase the number of samples to confirm the reliability of the results. The severity of these problems requires more sampling and comprehensive analysis in future research, and comprehensive analysis of gene diversity of different chromosomes should also be considered.

## Data Availability

The original contributions presented in the study are included in the article/Supplementary Material, further inquiries can be directed to the corresponding authors.
